# Deep Matrix Factorization Based on Convolutional Neural Networks for Image Inpainting

**DOI:** 10.3390/e24101500

**Published:** 2022-10-20

**Authors:** Xiaoxuan Ma, Zhiwen Li, Hengyou Wang

**Affiliations:** 1 School of Electrical and Information Engineering, Beijing University of Civil Engineering and Architecture, Beijing 100044, China; 2 School of Science, Beijing University of Civil Engineering and Architecture, Beijing 100044, China

**Keywords:** matrix completion, image inpainting, matrix factorization, deep learning, neural network

## Abstract

In this work, we formulate the image in-painting as a matrix completion problem. Traditional matrix completion methods are generally based on linear models, assuming that the matrix is low rank. When the original matrix is large scale and the observed elements are few, they will easily lead to over-fitting and their performance will also decrease significantly. Recently, researchers have tried to apply deep learning and nonlinear techniques to solve matrix completion. However, most of the existing deep learning-based methods restore each column or row of the matrix independently, which loses the global structure information of the matrix and therefore does not achieve the expected results in the image in-painting. In this paper, we propose a deep matrix factorization completion network (DMFCNet) for image in-painting by combining deep learning and a traditional matrix completion model. The main idea of DMFCNet is to map iterative updates of variables from a traditional matrix completion model into a fixed depth neural network. The potential relationships between observed matrix data are learned in a trainable end-to-end manner, which leads to a high-performance and easy-to-deploy nonlinear solution. Experimental results show that DMFCNet can provide higher matrix completion accuracy than the state-of-the-art matrix completion methods in a shorter running time.

## 1. Introduction

Matrix completion (MC) [1,2,3,4,5] aims to recover a matrix with missing matrix elements or incomplete data. It has been successfully applied to a wide range of signal processing and image analysis tasks, including collaborative filtering [6,7], image in-painting [8,9,10], image denoising [11,12], and image classification [13,14]. The MC methods assume that the original matrix is low rank and the missing elements of the matrix can be estimated based on rank minimization. It should be noted that the rank minimization problem is generally non-convex and NP-hard [15]. A typical approach to address this issue is to establish a convex approximation of the original non-convex objective function.

Existing approaches for solving the MC problem are mainly based on nuclear norm minimization (NNM) and matrix factorization (MF). The NNM approach [16,17,18] aims to minimize the sum of matrix singular values, which is a convex relaxation of the matrix rank. The nuclear norm minimization can be solved by singular value thresholding (SVT) algorithms [19], inexact increasing Lagrange multiplier (IALM) methods [16], and an alternating direction method (ADM) [17,20]. One major disadvantage of the NNM approach is that singular value decomposition (SVD) needs to be performed in each iteration of the optimization process, which has very high computational complexity when the matrix size is large. To avoid this problem, matrix factorization (MF), which does not need SVD, has been proposed by researchers to solve the MC problem [6,21,22,23]. Assuming that the rank of the original matrix is known, the MF method aims to decompose and approximate the matrix into a product of a thin matrix and a short matrix [21,24,25,26], and then reconstruct the missing element using this low-rank representation. Low-rank matrix fitting (LMaFit) [21] was one of the earliest MF methods. Although LMaFit is able to obtain an exact solution, it is sensitive to the rank estimation and cannot be globally optimized due to its non-convex formulation.

Both the NNM and the MF methods assume the low-rank property of the original matrix. Their performance degrades significantly when this property does not hold any more and the data are generated from a nonlinear latent variable model [10,27,28,29]. Recently, encouraged by the remarkable success of deep learning in many computer vision and machine learning tasks [30,31,32,33], researchers have explored the deep learning methods to nonlinear MC problems [28,29,30]. For example, the autoencoder-based collaborative filtering (AECF) approach [34] learns an autoencoder network to map the input matrix into a latent space and then reconstructs the matrix by minimizing the reconstruction error. The deep learning-based matrix complementation (DLMC) method [28] learns a stacked autoencoder network with with a nonlinear latent variable model. One major disadvantage of these deep learning-based methods is that they are unable to explore the global structure of the matrix, which degrades their performance in matrix completion, especially in image analysis where the global structure plays an important role in its restoration process.

In this paper, we propose a deep matrix factorization and completion network (DMFCNet) for matrix completion by coupling deep learning with traditional matrix completion methods. Our main idea is to use a neural network to simulate the iterative update of variables in the traditional matrix factorization process and learn the underlying relationship between input matrix data and the recovered output data after matrix completion in an end-to-end manner. We apply the proposed method to image in-painting to demonstrate its performance.

The main contributions of this paper can be summarized as follows.

(1) Compared with existing methods, our proposed method is able to address the nonlinear data model problem faced by the traditional MC methods. It is also able to address the global structure problem in existing deep learning-based MC methods.
(2) The proposed method can be pre-trained to learn the global image structure and underlying relationship between input matrix data with missing elements and the recovered output data. Once successfully trained, the network does not need to be optimized again in the subsequent image in-painting tasks, thereby providing a high-performance and easy-to-deploy nonlinear matrix completion solution.
(3) To improve the performance of the proposed method, a new algorithm for pre-filling the missing elements of the image is proposed. This new padding method performs global analysis of the matrix data to predict the missing elements as their initial values, which improves the performance of matrix completion and image in-painting.

The rest of the paper is organized as follows. Section 2 reviews related work. Section 3 presents our approach of deep matrix factorization and completion for image in-painting. Experimental results are presented in Section 4. Section 5 concludes the paper with a discussion of future research work.

## 2. Related Work

In this section, we review existing work related to our proposed method. For example, the mathematical models of the low-rank Hankel matrix factorization (LRHMF) method [35] and the deep Hankel matrix factorization (DHMF) method [36] will be introduced, respectively. The LRHMF method is a low-rank matrix factorization method that avoids singular value decomposition to achieve fast signal reconstruction. The DHMF method [36] inspired by LRHMF is a complex exponential signal recovery method based on deep learning and Hankel matrix factorization. The method proposed in this paper for image in-painting is inspired by them.

As said in [35], the rank of the Hankel matrix is equal to the number of exponentials in *x* which is a vector of exponential functions. Thus, the low-rank Hankel matrix completion (LRHMC) problem can be solved by using the low-rank property of the Hankel matrix. Its mathematical formula can be described as:

(1) $$\min_{x}{∥Rx∥}_{*}+\frac{\lambda }{2}{∥y-Ux∥}_{2}^{2}.$$

where *x* is the signal to be recovered from the undersampled data *y*, R is the operator that converts the signal to the Hankel matrix Rx, *U* denotes the undersampling matrix, and λ is the balance parameter. $\|\cdot {\|}_{*}$ is the nuclear norm of the matrix, which is used to restrict the rank of the matrix. The second term is used to measure the consistency of the data. However, it is very time-consuming to solve this problem because of its frequent singular value decomposition (SVD). To avoid this problem, the LRHMF method uses matrix factorization [37,38] instead of the nuclear norm minimization. Given any matrix, its nuclear norm can be approximated as:

(2) $${∥V∥}_{*}=\min_{P,Q}\frac{1}{2}\left({∥P∥}_{F}^{2}+{∥Q∥}_{F}^{2}\right),s.t.V=PQ^{H}.$$

where $P\in R^{n_{1}\times r}$, $Q\in R^{n_{2}\times r}$, ${\left\|\cdot \right\|}_{F}^{2}$ denotes the square of the Frobenius norm of the matrix, and the superscript *H* denotes the conjugate transpose. If we substitute Equation (2) to optimization problem (1), then the optimization problem can be reformulated as:

(3) $$\begin{array}{c} \begin{array}{c} \min_{x,P,Q}\frac{1}{2}\left({\left\|P\right\|}_{F}^{2}+{\left\|Q\right\|}_{F}^{2}\right)+\frac{\lambda }{2}{\left\|y-Ux\right\|}_{2}^{2},\\ s.t.Rx=PQ^{H}. \end{array} \end{array}$$

Since the nuclear norm of Rx is replaced by the Frobenius norm of its matrix factorization, it is no longer necessary to calculate the singular value decomposition. To solve this problem effectively, the alternating direction multiplier method (ADMM) is adopted in LRHMF [35], and its corresponding extended Lagrangian function is derived as:

(4) $$\begin{array}{c} \begin{array}{c} L\left(x,P,Q,D\right)=\frac{1}{2}{\left\|P\right\|}_{F}^{2}+\frac{1}{2}{\left\|Q\right\|}_{F}^{2}+\frac{\lambda }{2}{\left\|y-Ux\right\|}_{2}^{2}\\ +<D,Rx-PQ^{H}>+\frac{\gamma }{2}{∥Rx-PQ^{H}∥}_{F}^{2}. \end{array} \end{array}$$

where *D* denotes the increasing Lagrange multiplier, <·,·> denotes the inner product operator, and the λ>0 and γ>0 are the balanced parameters.

To solve the signal reconstruction problem, Huang et al. [36] gave the *k*-th iteration of the solution of (3) by minimizing (4), as shown in (5). Based on this iterative formulation, a deep Hankel matrix factorization network based on deep learning is designed for fast reconstruction of the signal.

(5) $$\begin{array}{c} \left\{\begin{array}{c} x^{k+1}={\left(\lambda U^{T}U+\gamma R^{*}R\right)}^{-1}\left(\lambda U^{T}y+\gamma R^{*}\left(P^{k}{\left(Q^{k}\right)}^{H}-D^{k}\right)\right)\\ P^{k+1}=\gamma \left(Rx^{k+1}+D^{k}\right)Q^{k}{\left(\gamma {\left(Q^{k}\right)}^{H}Q^{k}+I\right)}^{-1}\\ Q^{k+1}=\gamma \left(Rx^{k+1}+D^{k}\right)P^{k+1}{\left(\gamma {\left(P^{k+1}\right)}^{H}P^{k+1}+I\right)}^{-1}\\ D^{k+1}=D^{k}+\tau^{k}\left(Rx^{k+1}-P^{k+1}{\left(Q^{k+1}\right)}^{H}\right) \end{array}\right. \end{array}$$

## 3. The Proposed Method

In this section, we construct a deep matrix factorization completion network (DMFCNet) for matrix completion and image in-painting. We derive the mathematical model for our DMFCNet method, discuss the network design, and then introduce two network structures based on different prediction methods for missing elements. Finally, we introduce the loss functions and explain the network training process.

### 3.1. Mathematical Model of the DMFCNet Method

The proposed DMFCNet is based on low-rank matrix factorization [35,36]. The optimization objective function of our proposed model can be formulated as

(6) $$\begin{array}{c} \min_{X}{∥X∥}_{*}+\frac{\lambda }{2}{\left\|\Psi ⊙\left(Y-X\right)\right\|}_{F}^{2}, \end{array}$$

where $Y\in R^{m\times n}$ is the observation matrix with missing elements whose initial values are set to be a predefined constant. $X\in R^{m\times n}$ is the matrix that needs to be recovered from the matrix *Y*, and λ is a regularization parameter. $\|X{\|}_{*}$ is the nuclear norm of the matrix *X*, which is used to restrict the rank of *X*. ${\left\|\Psi ⊙\left(Y-X\right)\right\|}_{F}^{2}$ denotes the reconstruction error of *Y*, where ⊙ is the Hadamard product. $\Psi \in {\left\{0,1\right\}}^{m\times n}$ is a mask indicating the positions of missing data. If *Y* is missing data at position (i,j), the value of $\Psi_{ij}$ is 0; otherwise, it is 1. We use matrix factorization instead of the traditional nuclear norm minimization, and the proposed model can be formulated as follows:

(7) $$\begin{array}{c} \begin{array}{c} \min_{X,U,V}\frac{1}{2}\left({\left\|U\right\|}_{F}^{2}+{\left\|V\right\|}_{F}^{2}\right)+\frac{\lambda }{2}{\left\|\Psi ⊙\left(Y-X\right)\right\|}_{F}^{2},\\ s.t.\;X=UV^{T}, \end{array} \end{array}$$

where $U\in R^{m\times r}$ and $V\in R^{n\times r}$. The augmented Lagrangian function for (7) is given by

(8) $$\begin{array}{c} \begin{array}{c} L\left(X,U,V,S\right)=\frac{1}{2}{\left(\|U\right\|}_{F}^{2}+{\left\|V\right\|}_{F}^{2})+\frac{\lambda }{2}{\left\|\Psi ⊙\left(Y-X\right)\right\|}_{F}^{2}\\ +<S,X-UV^{T}>+\frac{\eta }{2}{∥X-UV^{T}∥}_{F}^{2}. \end{array} \end{array}$$

Here, η>0 is the penalty parameter and $S\in R^{m\times n}$ is the Lagrangian multiplier corresponding to the constraint $X=UV^{T}$. Since it is difficult to solve for *U*, *V*, *S* and *X* simultaneously in (8), following the idea of alternating direction method of multipliers (ADMM), we minimize the Lagrangian function with respect to each block variable *U*, *V*, *S* and *X* at a time while fixing the other blocks at their latest values. Thus, the proposed optimization process becomes:

(9) $$\begin{array}{c} \left\{\begin{array}{c} U_{k+1}=\underset{U\in R^{m\times r}}{\arg\min}L\left(X_{k},U_{k},V_{k},S_{k}\right),\\ V_{k+1}=\underset{V\in R^{n\times r}}{\arg\min}L\left(X_{k},U_{k+1},V_{k},S_{k}\right),\\ S_{k+1}=S_{k}+\mu \left(X_{k}-U_{k+1}{\left(V_{k+1}\right)}^{T}\right),\\ X_{k+1}=\underset{X\in R^{m\times n}}{\arg\min}L\left(X_{k},U_{k+1},V_{k+1},S_{k+1}\right), \end{array}\right. \end{array}$$

where μ>0 is the step size of the optimization process.

**Figure 1:**
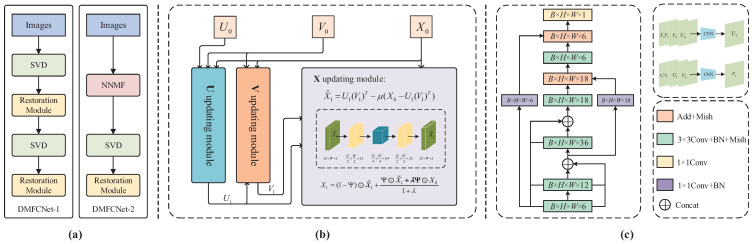
The structure of the DMFCNet network. (**a**) Network architecture. (**b**) Restoration Module. (**c**) **U** and **V** updating modules.

However, there are many limitations if solved directly by traditional algorithms, so we propose to solve the above optimization problem using a deep learning approach. The main idea is to update the variables using neural network modules. As shown in Figure 1, we construct a deep neural network based on (9), which has three updating modules shown in Figure 1b. A completed restoration module contains the **U** updating module and **V** updating module for updating matrices *U* and *V*, and it contains the **X** updating module for restoring the incomplete matrix.

#### 3.1.1. U and V Updating Modules

In our proposed DMFCNet method, the input matrix is first processed by **U** and **V** updating modules. According to the analysis in [36], *U* and *V* are updated as follows:

(10) $$\begin{array}{c} \left\{\begin{array}{c} U_{1}=\eta \left(X_{0}+S_{0}\right)V_{0}{\left(\eta {V_{0}}^{T}V_{0}+I\right)}^{-1},\\ V_{1}=\eta {\left(X_{0}+S_{0}\right)}^{T}U_{1}{\left(\eta {U_{1}}^{T}U_{1}+I\right)}^{-1}. \end{array}\right. \end{array}$$

Note that the variables $\left(X_{0}+S_{0}\right)V_{0}$ and $V_{0}$ are included in the update formula of *U*. So, we choose to add them to the input of the **U** updating module. In order to learn the maximum convolutional features, $U_{0}$ is also added as an input to the **U** updating module. The auxiliary matrix variable $S_{0}$ in (10) is initialized as a zero matrix; thus, it can be removed in the **U** and **V** updating modules. Based on (10), for the **U** updating module, we concatenate variables $X_{0}V_{0}$, $V_{0}$ and $U_{0}$ in the channel dimension as input and use a convolutional neural network to update the variable. The updating of the *V* matrix follows a similar procedure.

Once $U_{1}$ is updated, we concatenate ${X_{0}}^{T}U_{1}$, $U_{1}$ and $V_{0}$ channel-wise to obtain $V_{1}$. Thus, the updating formulas for *U* and *V* matrices are:

(11) $$\begin{array}{c} \left\{\begin{array}{c} U_{1}=C_{U}\left(X_{0}V_{0},V_{0},U_{0}\right),\\ V_{1}=C_{V}\left({X_{0}}^{T}U_{1},U_{1},V_{0}\right), \end{array}\right. \end{array}$$

where C denotes the convolutional neural network.

We observe that the final matrix recovery performance is sensitive to the initialization of *U* and *V*. To address this issue, we propose to perform the following SVD of $X_{0}\in R^{m\times n}$ to initialize *U* and *V*:

(12) $$\begin{array}{c} X_{0}=U\Sigma V^{T},\Sigma =diag\left({\left\{{\tilde{\sigma }}_{i}\right\}}_{1\leq i\leq d}\right). \end{array}$$

where $\Sigma \in R^{d\times d}$ is a diagonal matrix with ${\tilde{\sigma }}_{1},\ldots ,{\tilde{\sigma }}_{d}$ on the diagonal and zeros elsewhere, d=min(m,n). ${\tilde{\sigma }}_{i}>0$ is the *i*-th singular value of matrix $X_{0}$. $U\in R^{m\times d}$ and $V\in R^{n\times d}$ are left and right singular vectors, respectively. Then, $U_{0}\in R^{m\times r}$ and $V_{0}\in R^{n\times r}$ are initialized by

(13) $$\begin{array}{c} U_{0}=\tilde{U}\sqrt{\tilde{\Sigma }},V_{0}=\tilde{V}\sqrt{\tilde{\Sigma }}, \end{array}$$

where $\tilde{U}\in R^{m\times r}$ are the first *r* columns of *U*, $\tilde{V}\in R^{n\times r}$ are the first *r* columns of *V*, and $\tilde{\Sigma }\in R^{r\times r}$ are the first *r* rows and first *r* columns of Σ. In this paper, we set m=n.

In order to maintain the maximum amount of information during the matrix completion process, a dense convolutional structure is used in the network, and a residual structure is added to improve the stability of the training process. The Mish function is chosen as the activation function due to its smoothness at almost all points of the curve, which allows more information to flow through the neural network. A batch normalization operation (BN) layer is added between convolution layers to speed up the convergence.

#### 3.1.2. X Updating Module

After obtaining $U_{1}$ and $V_{1}$ using the **U** and **V** updating modules, the Lagrange multiplier $S_{1}$ can be updated using following formula:

(14) $$\begin{array}{c} S_{1}=\mu \left(X_{0}-U_{1}{V_{1}}^{T}\right) \end{array}$$

Then, they will be fed into the **X** updating module, and ${\hat{X}}_{1}$ will be obtained by the following equation.

(15) $$\begin{array}{c} {\hat{X}}_{1}=U_{1}{V_{1}}^{T}-S_{1}. \end{array}$$

To improve the reconstruction performance, we further process ${\hat{X}}_{1}$ by an autoencoder network. As shown in Figure 1b, the network contains four convolution layers, a batch normalization module, and the last activation layer with the tanh function. For image in-painting applications, to enhance the smoothness of the recovered image, we incorporate the following weighted averaging operation into the network

(16) $$\begin{array}{c} X_{1}=\left(1-\Psi \right)⊙{\tilde{X}}_{1}+\frac{\Psi ⊙{\tilde{X}}_{1}+\gamma \Psi ⊙X_{0}}{1+\gamma }. \end{array}$$

where $X_{0}$ is the initial matrix and γ is a weighting parameter. When a pixel value is missing at a point in the image, the output of the network is assigned directly to the value at the corresponding location. Otherwise, a weighted average between the output of the network and the pixel value of the corresponding location of the input image are used to obtain the final reconstructed pixel value of that location.

### 3.2. Pre-Filling

Note that the initialization of *U* and *V* in the network is obtained from the original incomplete matrix using SVD, so the missing entries in the matrix need to be filled with predefined constants before the singular value decomposition. However, the network is extremely sensitive to the pre-filled constants and will directly affect the in-painting performance if not filled properly.

To reduce the effect of filling random constants, we first obtain $X_{0}$ by replacing the missing values of the observation matrix with predefined constants such as 255. Then, the singular value decomposition operation is performed on $X_{0}$ to obtain $U_{0}$ and $V_{0}$ and input them into the restoration module for preliminary restoration to obtain $X_{1}$, and the output matrix $X_{new}$ can be calculated by:

(17) $$X_{new}=\left(1-\Psi \right)⊙X_{1}+\Psi ⊙X_{0}.$$

This step is the preliminary inference of the missing values by the restoration module. The predicted values are filled to the missing positions of the observation matrix, and then, the filled $X_{new}$ is used as the input to the second restoration module. Since $U_{0}$ and $V_{0}$ in the second restoration module are obtained by the singular value decomposition of the new $X_{0}$, which can largely eliminate the negative effects of using random constant filling, better restoration results can be obtained by the second restoration module. The following Algorithm 1 summarizes the DMFCNet-1 algorithm for network-based pre-filling operations.
**Algorithm 1** DMFCNet-1**Require**: $X_{\Omega }$: original incomplete image matrix; Ω: the position of the observed entries; non-negative parameters *r*, μ and λ.**Ensure:** the restored matrix $X_{new}$.  1:Init: $X_{0}\in R^{m\times n}$: The matrix using constant 255 to replace missing values of $X_{\Omega }$; $\Psi \in {\left\{0,1\right\}}^{m\times n}:\Psi_{ij}=\left\{\begin{array}{c} 1,if(i,j)\in \Omega \\ 0,if(i,j)\notin \Omega \end{array}\right.$;  2:**for***i* = 1:2 **do**  3:   **Compute** $U_{0}\in R^{m\times r}$ and $V_{0}\in R^{n\times r}$ from $X_{0}$ using (12) and (13);  4:   $U_{1}\leftarrow C_{U}\left(X_{0}V_{0},V_{0},U_{0}\right)$;  5:   $V_{1}\leftarrow C_{V}\left({X_{0}}^{T}U_{1},U_{1},V_{0}\right)$;  6:   ${\hat{X}}_{1}\leftarrow U_{1}{V_{1}}^{T}-\mu \left(X_{0}-U_{1}{V_{1}}^{T}\right)$;  7:   ${\tilde{X}}_{1}\leftarrow \mathrm{AutoEncoder}({\hat{X}}_{1})$;  8:   $X_{1}\leftarrow \left(1-\Psi \right)⊙{\tilde{X}}_{1}+\frac{\Psi ⊙{\tilde{X}}_{1}+\lambda \Psi ⊙X_{0}}{1+\lambda }$;  9:   $X_{new}\leftarrow \left(1-\Psi \right)⊙X_{1}+\Psi ⊙X_{0}$;  10: $X_{0}\leftarrow X_{new}$;  11:**end for**  12:**return**$X_{new}$;

However, a pre-filling-based neural network requires a singular value decomposition, which will take a relatively long time. To improve the running time, according to the structural characteristics of the image, a new pre-filling algorithm, called Nearest Neighbor Mean Filling (NNMF), is presented. It takes the data observed near the location of the missing value as a reference to infer the missing value.

It is assumed that we need to fill the data at the location (i,j) of the missing values. Let $V_{lij}$ be the value of the first non-missing position traversed from position (i,j) to the left, $V_{rij}$ be the value of the first non-missing position traversed from position (i,j) to the right, and similarly, let $V_{tij}$ be the value of the first non-missing position traversed from position (i,j) to the top and $V_{bij}$ be the value of the first non-missing position traversed from position (i,j) to the bottom. Then, the formula for filling the data $V_{ij}$ at the location (i,j) of the missing value is as follows.

(18) $$V_{ij}=\frac{\left(V_{lij}+V_{rij}+V_{tij}+V_{bij}\right)}{4}.$$

**Figure 2:**
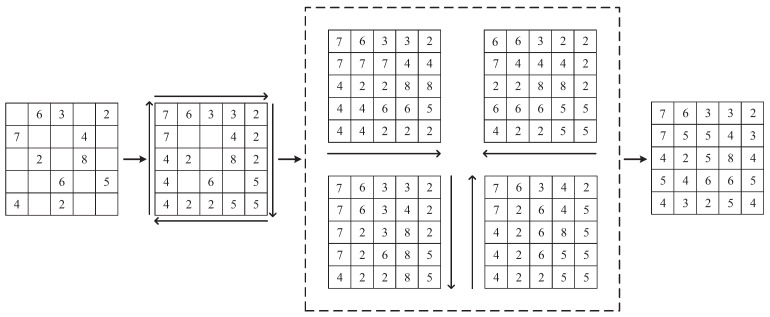
Schematic diagram of the operation of the NNMF algorithm.

It is a very time-consuming operation to traverse the location of each missing value and then find the four values in turn. In this paper, we design a calculation procedure as shown in Figure 2, which can efficiently calculate the fill values at the locations of all missing data by dynamic programming. As shown in Figure 2, the missing values at the edges are first filled in a clockwise direction; then, four matrices are generated in four directions, and finally, the four generated matrices are summed to find the mean value to obtain the filled matrix.

Algorithm 2 summarizes the DMFCNet-2 algorithm based on NNMF for pre-filling operation. The matrix obtained from the pre-filling operation of the observation matrix using the NNMF algorithm is used as the input of the restoration module. Based on the two pre-filling methods, the network framework of the DMFCNet-1 algorithm and DMFCNet-2 algorithm proposed in this paper is shown in Figure 1a. During training, only the weighting parameters in the convolutional networks $C_{U}$ and $C_{V}$ and the autoencoder are optimized.
**Algorithm 2** DMFCNet-2**Require:** $X_{\Omega }$: original incomplete image matrix; Ω: the position of the observed entries; non-negative parameters *r*, μ and λ.**Ensure:** the restored matrix $X_{new}$.  1:Init: $X_{0}\in R^{m\times n}$: The matrix obtained by pre-filling $X_{\Omega }$ using NNMF algorithm; $\Psi \in {\left\{0,1\right\}}^{m\times n}:\Psi_{ij}=\left\{\begin{array}{c} 1,if(i,j)\in \Omega \\ 0,if(i,j)\notin \Omega \end{array}\right.$;  2:**Compute**$U_{0}\in R^{m\times r}$ and $V_{0}\in R^{n\times r}$ from $X_{0}$ using (12) and (13);  3:$U_{1}\leftarrow C_{U}\left(X_{0}V_{0},V_{0},U_{0}\right)$;  4:$V_{1}\leftarrow C_{V}\left({X_{0}}^{T}U_{1},U_{1},V_{0}\right)$;  5:${\hat{X}}_{1}\leftarrow U_{1}{V_{1}}^{T}-\mu \left(X_{0}-U_{1}{V_{1}}^{T}\right)$;  6:${\tilde{X}}_{1}\leftarrow \mathrm{AutoEncoder}({\hat{X}}_{1})$;  7:$X_{1}\leftarrow \left(1-\Psi \right)⊙{\tilde{X}}_{1}+\frac{\Psi ⊙{\tilde{X}}_{1}+\lambda \Psi ⊙X_{0}}{1+\lambda }$;  8:$X_{new}\leftarrow \left(1-\Psi \right)⊙X_{1}+\Psi ⊙X_{0}$;  9:**return**$X_{new}$;

### 3.3. Loss Function

The general convolutional neural network, whose network interior is equivalent to a black box for people, can only be globally optimized by constraining the final output of the network to the whole network weights. In contrast, each variable in the interpretable network built based on the iterative model in this paper is of practical significance. So, in addition to restricting the final output $X_{1}$ of the restoration module in the loss function, this paper also restricts the intermediate variables in the module, which can make its training more stable and efficient. Frobenius parametrization is used to restrict the variables in the network, from which the loss function of a recovery module can be derived as follows:

(19) $$\begin{array}{c} \begin{array}{c} L\left(\Theta \right)=\frac{1}{B}\sum_{b=1}^{B}(\left({∥X_{b}\left(\Theta \right)-Y_{b}∥}_{F}^{2}\right)+\alpha \left({∥{\hat{X}}_{b}\left(\Theta \right)-Y_{b}∥}_{F}^{2}\right)\\ +\beta ({∥U_{b}\left(\Theta \right)V_{b}{\left(\Theta \right)}^{T}-Y_{b}∥}_{F}^{2})). \end{array} \end{array}$$

where Θ is the network parameter of the restoration module, *B* is the number of samples input to the network, and α and β are the regular term coefficients. $X_{b}$ denotes the output $X_{1}$ of the *b*-th sample in the restoration module and ${\hat{X}}_{b}$ is the input ${\hat{X}}_{1}$ of the *b*-th sample of the autoencoder in the **X** updating module. $U_{b}$ and $V_{b}$ are the $U_{1}$ and $V_{1}$ of the *b*-th sample output, and $Y_{b}$ is the complete image corresponding to the *b*-th sample.

### 3.4. Training

According to the diversity of the VOC dataset [39,40], this dataset is selected as the training sample to adapt to the recovery task of more complex images. Firstly, the image is converted into a grayscale image of size 256 × 256, and then, some random pixel values in the image are replaced by 255.

The hyperparameters in training are set as follows. The first 50 singular values are taken when initializing the *U* and *V* matrices. Adam is chosen as the optimizer for training the network, and the learning rate is set to $1\times 10^{-3}$, which is reduced to $1\times 10^{-4}$ after stabilization and set to $1\times 10^{-5}$ for global fine-tuning. μ is set to $1\times 10^{-3}$ and γ is set to 10 in the **X** updating module. The loss function canonical term coefficients α and β are set to 0.1 and 0.01, respectively. The autoencoder in the **X** updating module contains a total of three hidden layers with the dimensions $\left(\frac{H}{2},\frac{W}{2},32\right)$, $\left(\frac{H}{4},\frac{W}{4},64\right)$ and $\left(\frac{H}{2},\frac{W}{2},32\right)$.

To make it more targeted for the recovery of images with missing elements, two models are trained for each of the DMFCNet-1 and DMFCNet-2 networks. The first model uses a dataset containing images with a 30% to 50% missing rate, so this model is mainly used for recovering images with a 50% missing rate and below. The second model uses a dataset containing images with a 50% to 70% missing rate, and this model is used to recover images with a 50% to 70% missing rate.

**Figure 3:**
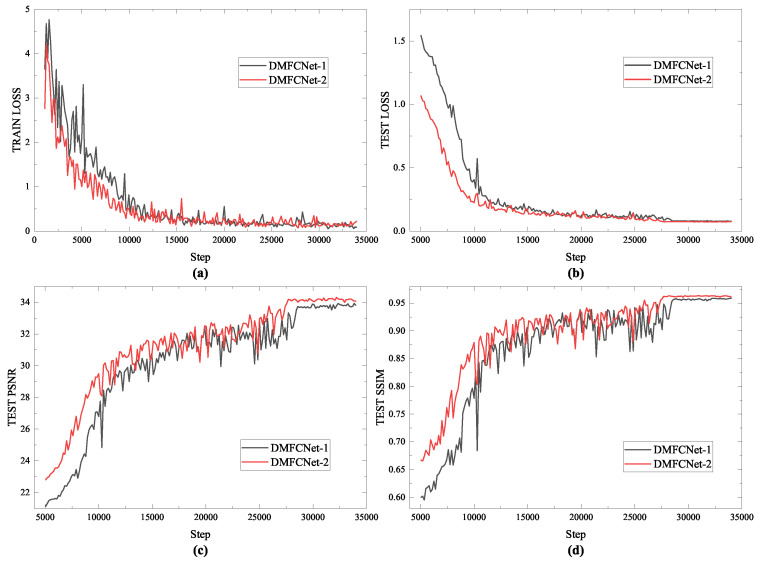
The loss convergence of the network and the reconstruction results. (**a**) Loss convergence of the training. (**b**) Loss convergence of the test set. (**c**) PSNR values of the reconstructed images in the test set. (**d**) SSIM values of the reconstructed images in the test set.

Specifically, DMFCNet-1 is trained with one restoration module as the training unit. The first restoration module is trained, and the weights of the first restoration module are frozen after the training is completed. Then, the second restoration module is added and trained, and the weights of the first repair module are unfrozen for global fine-tuning when the training of the second restoration module is completed. Figure 3 shows the loss convergence during the training period of the two models and the reconstruction results of the test data.

## 4. Experiments

In this section, we first compare the two versions of the DMFCNet model proposed in this paper in image in-painting tasks, and then, we compare them with six popular matrix completion methods. These methods are matrix factorization (MF) by LmaFit [21], nuclear norm minimization (NNM) by IALM [16], truncated nuclear norm minimization (TNNM) by ADMM [8], DLMC method-based deep learning [28], NC-MC method [41], and LNOP method by ADMM [42]. The peak signal-to-noise ratio (PSNR) [43] and structural similarity (SSIM) [44] were used in the experiments to evaluate the quality of the restored images.

### 4.1. Datasets

In this part, we discuss how to select the dataset for training the model. The method proposed requires pre-training the network model parameters, which requires a large number of datasets for training. We hope that the proposed algorithm is not only limited to simple low-rank images but includes both low-rank images and more complex images. Therefore, two datasets are chosen to train the model and test the effect of different datasets on the image restoration performance. The first dataset is the CelebFaces Attributes Dataset (CelebA) [45], which is a large-scale face attribute dataset with over 200,000 celebrity images. These images contain some degree of pose variation but remain relatively simple and homogeneous images overall. The second dataset is the VOC dataset [39,40], which has a more diverse set of images, including simple low-rank images as well as complex images.

**Figure 4:**
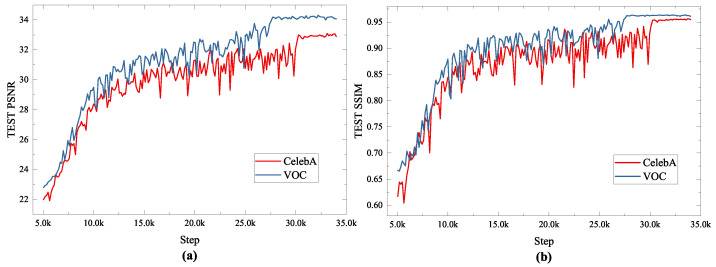
Test results of the model on complex images obtained by training with different datasets. (**a**) Average PSNR values of recovered images (**b**) Average SSIM values of recovered images.

Two datasets are used to train the DMFCNet-2 model, where the images of the datasets are converted to grayscale images of size 256 × 256, and 30% of random pixel information will be discarded. The test results of the model on complex images obtained by training with different datasets are shown in Figure 4. It can be seen from Figure 4 that the training loss when training the network using the CelebA dataset is smaller than that when training the network using the VOC dataset because of its relative simplicity. However, the loss and reconstruction performance of the network trained with the VOC dataset outperformed the network trained with the CelebA dataset when tested on complex images. Therefore, to improve the image restoration performance, we recommend using a more targeted dataset.

### 4.2. Experimental Settings

To make the best performance of six methods for comparison, the hyperparameters of each method were chosen as follows. In MF, since automatic estimation often leads to poor performance in image restoration problems, the fixed number of ranks is chosen for different missing rates, with the rank set to 30 for restoring images containing 20% to 30% missing rates, 20 for restoring images containing 40% to 50% missing rates, and 10 for restoring images containing 60% to 70% missing rates and text masks. In TNNM, the parameter *r* is uniformly set to 10. In DLMC, the weight decay penalty is set to 0.01, the network contains three hidden layers, and the number of hidden cells is set to [100 50 100]. *p* is set to 0.7 in LNOP. Other parameters follow the settings in the original paper.

### 4.3. Image In-Painting

**Figure 5:**
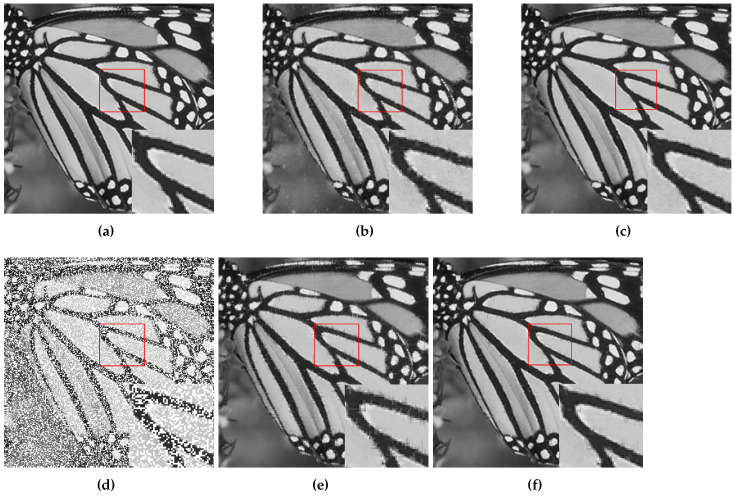
DMFCNet-1 and DMFCNet-2 restore image containing 40% missing rate. (**a**) Original image. (**b**) Preliminary restoration result of DMFCNet-1. (**c**) Final restoration result of DMFCNet-1. (**d**) Partially missing image. (**e**) Preliminary restoration result of DMFCNet-2. (**f**) Final restoration result of DMFCNet-2.

**Figure 6:**
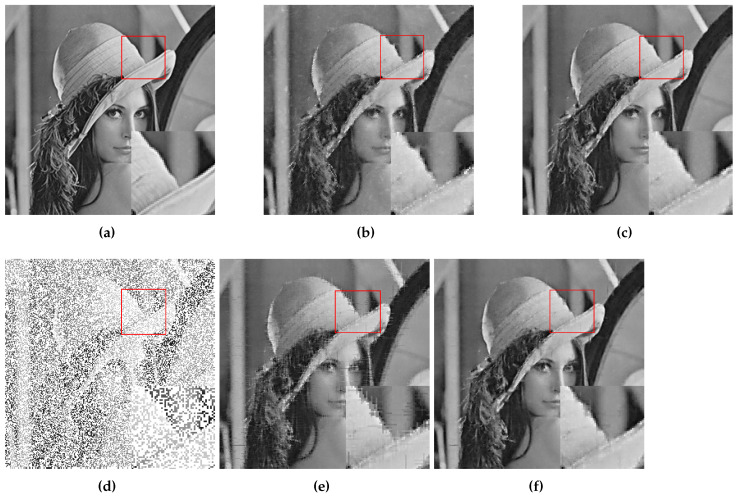
DMFCNet-1 and DMFCNet-2 restore image containing 60% missing rate. (**a**) Original image. (**b**) Preliminary restoration result of DMFCNet-1. (**c**) Final restoration result of DMFCNet-1. (**d**) Partially missing image. (**e**) Preliminary restoration result of DMFCNet-2. (**f**) Final restoration result of DMFCNet-2.

At first, DMFCNet-1 and DMFCNet-2 are compared, which includes the preliminary restoration results (pre-filling results) and the final restoration results. Figure 5 and Figure 6 show the restoration results of the two models restoring images containing 40% and 60% missing rates. As shown in Figure 5e and Figure 6e, the image pre-filled with NNMF can achieve relatively good results in the relatively smooth areas of the image, but it produces more obvious vertical stripes in the areas with large variations in pixel values. Figure 5f and Figure 6f show that the vertical stripes of the restored image using the DMFCNet-2 model have disappeared a lot, and the overall image is smoother, but there are still some spots left by the pre-filling of the image with NNMF. The DMFCNet-1 model uses the restoration module for the preliminary restoration, as shown in Figure 5b and Figure 6b. As can be seen, although there is no vertical stripe, the image has some white spots and is rougher overall. Figure 5c and Figure 6c show the final restoration result of DMFCNet-1.

It can be seen that after the second restoration module, the image was restored more carefully based on the preliminary restoration. The white spots in the image basically disappear, but the overall image is a bit rougher than the restored result of DMFCNet-2. In addition, Table 1 shows the recovery of the two methods at different missing rates, which contains a performance comparison of the preliminary restoration ability of the two models. It can be seen from Table 1 that the DMFCNet-2 model with pre-filling using NNMF is better at a low missing rate, but the preliminary restoration of DMFCNet-1 at a high missing rate gives stronger results than that of pre-filling using NNMF. However, the recovery of DMFCNet-2 is better than that of DMFCNet-1 because the images obtained by NNMF are smoother overall.

**Figure 7:**
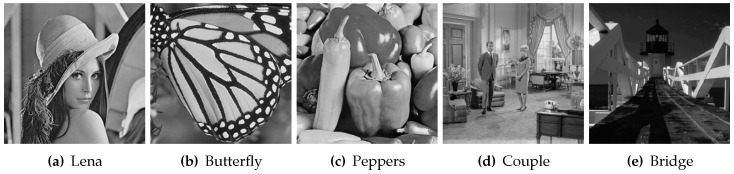
Five grayscale images of 256 × 256 size for comparison experiments, numbered 1–5 from left to right.

The next step is to compare the proposed methods with other methods of matrix completion. Five images as shown in Figure 7 are selected for the comparison experiments. Two masks are considered in the experiments: the first one is a random pixel mask, where 20% to 70% of the pixels in the image are removed randomly. The second one is a text mask containing English words. Although the DMFCNet-1 model and DMFCNet-2 model are not trained to restore images that contain text masks, the DMFCNet-2 network is used to compare with other methods in the tests containing text masks because of the characteristics of NNMF.

**Figure 8:**
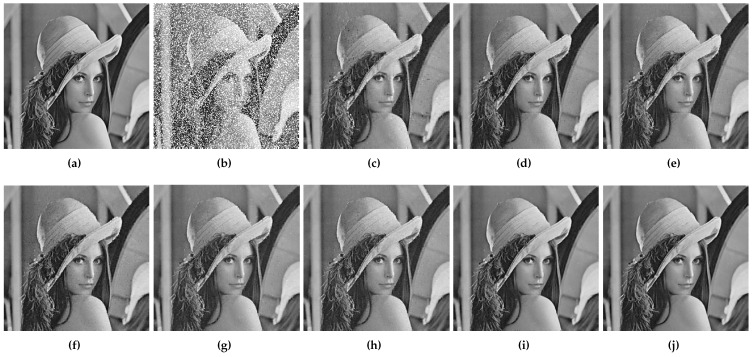
Image recovery containing a 30% random pixel mask. (**a**) Complete image of size 256 × 256. (**b**) Partially missing image (10.52 dB/0.127). (**c**) Restored result by MF in 0.099 s (27.42 dB/0.822/0.099 s). (**d**) Restored result by NNM (29.37 dB/0.873/5.488 s). (**e**) Restored result by TNNM (29.676 dB/0.883/2.669 s). (**f**) Restored result by DLMC (29.354 dB/0.867/15.181 s). (**g**) Restored result by NC-MC (29.69 dB/0.893/5.399 s). (**h**) Restored result by LNOP (29.816 dB/0.881/2.391 s). (**i**) Restored result by DMFCNet-1 (33.29 dB/0.956/0.388 s). (**j**) Restored result by DMFCNet-2 (34.68 dB/0.971/0.345 s).

**Figure 9:**
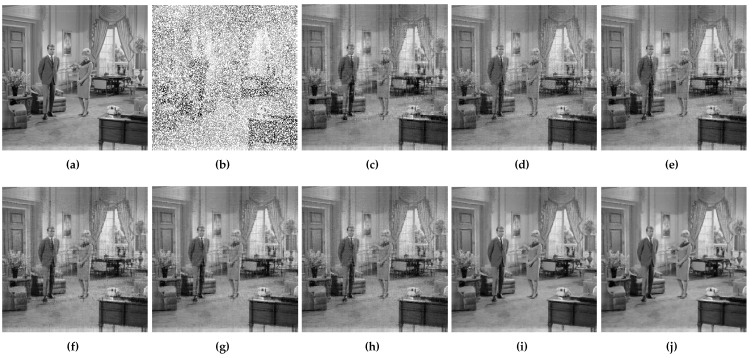
Image recovery containing a 50% random pixel mask. (**a**) Complete image of size 256 × 256. (**b**) Partially missing image (8.29 dB/0.076). (**c**) Restored result by MF (25.69 dB/0.784/0.072 s). (**d**) Restored result by NNM (26.82 dB/0.805/4.297 s). (**e**) Restored result by TNNM (27.264 dB/0.820/3.094 s). (**f**) Restored result by DLMC (27.08 dB/0.812/17.956 s). (**g**) Restored result by NC-MC (27.63 dB/0.837/3.473 s). (**h**) Restored result by LNOP (27.29 dB/0.816/2.105 s). (**i**) Restored result by DMFCNet-1 (29.73 dB/0.898/0.403 s). (**j**) Restored result by DMFCNet-2 (30.04 dB/0.908/0.346 s).

**Figure 10:**
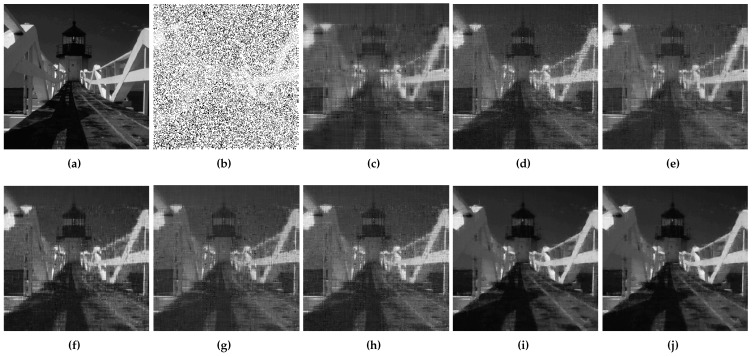
Image recovery containing a 70% random pixel mask. (**a**) Complete image of size 256 × 256. (**b**) Partially missing image (4.61 dB/0.024). (**c**) Restored result by MF (24.86 dB/0.614/0.034 s). (**d**) Restored result by NNM (25.78 dB/0.683/4.836 s). (**e**) Restored result by TNNM (26.126 dB/0.664/8.630 s). (**f**) Restored result by DLMC (27.057 dB/0.719/19.767 s). (**g**) Restored result by NC-MC (26.09 dB/0.663/2.266 s). (**h**) Restored result by LNOP (26.53 dB/0.68/1.808 s). (**i**) Restored result by DMFCNet-1 (29.82 dB/0.859/0.449 s). (**j**) Restored result by DMFCNet-2 (31.47 dB/0.884/0.339 s).

**Figure 11:**
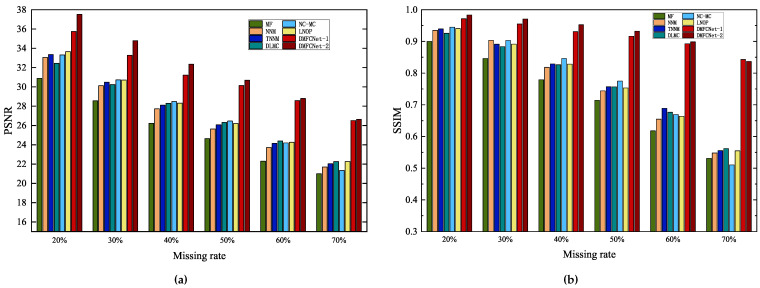
The average restoration performance of the eight methods for five images with different missing rates. The PSNR and SSIM values of the recovered images are shown on the (**a**) and (**b**), respectively.

**Figure 12:**
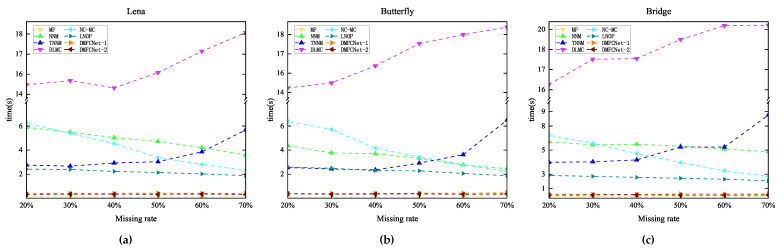
Execution times of eight methods to recover grayscale images of size 256 × 256 containing different missing rates. (**a**) Lena. (**b**) Butterfly. (**c**) Bridge.

Figure 8, Figure 9 and Figure 10 show the original images, images containing random pixel masks, and examples of restored images obtained by each of the six methods. Here, 30%, 50% and 70% of the pixels in the original image are removed randomly, respectively. From the images, it can be visually seen that the images obtained by restoration through the MF method are rougher than those obtained by other methods, and DMFCNet-1 and DMFCNet-2 have the best restoration results. We also conducted more comprehensive tests on other images, and the experimental results are shown in Table 2. Table 2 shows the PSNR values and SSIM values of the images obtained from the recovery of five images containing a 20% to 70% missing rate by six methods, respectively. Figure 11 illustrates the average recovery performance for five images with different missing rates using the eight methods. Figure 12 shows the execution time of the eight methods to recover grayscale images of size 256 × 256 containing different missing rates. Meanwhile, Table 3 shows the average running times of the eight methods for recovering images of different sizes containing different missing rates.

The graphical data show that MF(LMaFit) takes the shortest time, which is followed by the methods proposed in this paper. Due to the superiority of deep learning, using the trained network model for image restoration can significantly reduce the time required for the restoration task; even if the missing rate increases gradually, it does not increase the time required for restoration. In contrast, the deep learning-based DLMC method takes the longest time because it needs to optimize the network weights, and its running time growth rate is the largest among all methods when the image size increases. From the overall graphical data, it can be seen that DMFCNet-1 and DMFCNet-2 can achieve better recovery performance than competing methods in the shortest time, both for images containing small and large missing rates. Especially when the missing rate is large, the recovery performance of other methods decreases faster, but the proposed methods can still achieve satisfactory results.

**Figure 13:**
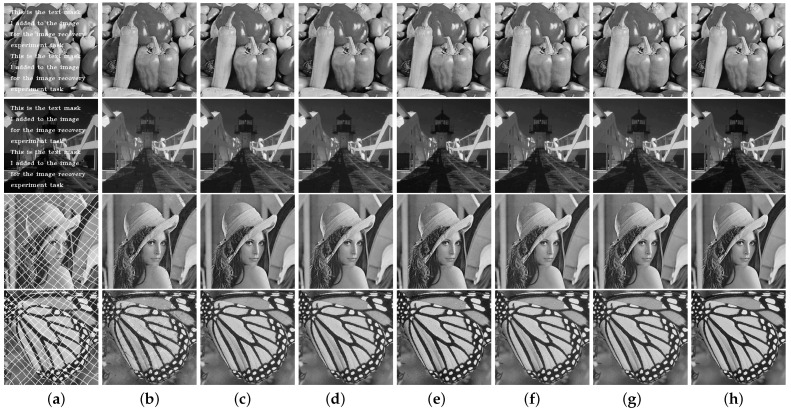
Image recovery with text mask and grid mask. (**a**) Images with masks. (**b**) Restored results by MF. (**c**) Restored results by NNM. (**d**) Restored results by TNNM. (**e**) Restored results by DLMC. (**f**) Restored results by NC-MC. (**g**) Restored results by LNOP. (**h**) Restored results by DMFCNet-2.

Figure 13 shows the examples of the images containing text masks and grid masks and the recovered images obtained by the seven methods. Table 4 shows the restoration results of the seven methods on the five images containing text masks and grid masks. The data in Table 4 show that the proposed DMFCNet-2 network, even though it is not trained to recover in the case of text-masked and grid-masked images, still performs well due to the characteristics of NNMF.

## 5. Conclusions

In this work, a new end-to-end neural network structure for image restoration called DMFCNet is proposed in this paper by combining deep learning with traditional matrix complementation algorithms. Experimental results on data containing random masks and other masks show that DMFCNet performs optimally in image restoration compared to the currently popular methods, and it remains stable even when it contains high missing rates.

Although the methods have good performance, there is still room for further improvement. For example, when restoring images containing a high missing rate, the restoration result of DMFCNet-1 contains white spots and the restoration result of DMFCNet-2 contains vertical stripes. Therefore, how to combine these two restoration results to obtain better restoration results is a problem that needs to be investigated in the future. In addition, the adjustment of hyperparameters in this method is also one of the important elements of the next work. A variety of other experiments will be carried out in the future in order to apply the methods of this paper to a wider range of experiments, such as larger image sizes (e.g., 512 × 512 pixels) or missing data due to other factors (e.g., image processing or transmission).

## Figures and Tables

**Table 1:** Restoration results of two methods of restoration containing different missing rates, each method contains preliminary restoration results (left) and final restoration results (right).

Missing Rate	Images NO.	PSNR/SSIM			
		DMFCNet-1		DMFCNet-2	
30%	1	29.89/0.904	33.29/0.956	31.11/0.952	34.68/0.971
	2	27.63/0.915	30.59/0.958	28.08/0.953	31.94/0.974
	3	30.80/0.908	33.44/0.959	32.21/0.963	35.29/0.973
	4	30.10/0.902	32.56/0.946	31.92/0.946	33.70/0.960
	5	32.52/0.875	36.47/0.956	35.59/0.968	38.32/0.976
	Average	30.19/0.901	33.27/0.955	31.78/0.956	34.78/0.971
50%	1	27.46/0.847	30.04/0.914	27.24/0.879	30.30/0.929
	2	23.98/0.863	27.23/0.929	23.24/0.856	27.74/0.939
	3	27.97/0.852	30.19/0.921	27.77/0.896	31.00/0.938
	4	27.56/0.831	29.73/0.898	28.18/0.873	30.04/0.908
	5	29.85/0.804	33.54/0.919	31.58/0.923	34.39/0.947
	Average	27.37/0.840	30.14/0.916	27.60/0.885	30.69/0.932
70%	1	24.92/0.763	26.23/0.834	23.79/0.729	26.12/0.829
	2	21.65/0.781	23.43/0.860	19.12/0.665	22.29/0.810
	3	25.02/0.770	26.62/0.861	23.94/0.744	26.66/0.853
	4	24.93/0.721	26.47/0.801	24.95/0.739	26.62/0.808
	5	27.70/0.746	29.82/0.859	28.14/0.832	31.47/0.884
	Average	24.84/0.756	26.51/0.843	23.99/0.742	26.63/0.837

**Table 2:** PSNR and SSIM values of the five images recovered by eight methods containing 20% to 70% missing rate, respectively. The best results are highlighted in bold.

Missing Rate	Images NO.	PSNR/SSIM							
		MF [21]	NNM [16]	TNNM [8]	DLMC [28]	NC-MC [41]	LNOP [42]	DMFCNet-1	DMFCNet-2
20%	1	29.72/0.890	32.06/0.929	32.30/0.935	31.22/0.913	32.05/0.939	32.55/0.934	35.60/0.972	**37.57**/**0.984**
	2	26.10/0.831	29.24/0.896	29.60/0.904	29.09/0.888	29.85/0.912	30.12/0.906	33.35/0.972	**35.03**/**0.985**
	3	30.90/0.901	33.26/0.939	33.54/0.943	32.70/0.933	33.37/0.948	33.92/0.945	35.77/0.973	**37.69**/**0.984**
	4	31.99/0.938	33.39/0.951	33.63/0.954	32.26/0.938	33.73/0.959	33.81/0.955	34.97/0.969	**36.20**/**0.978**
	5	35.71/0.939	37.31/0.959	37.71/0.961	36.92/0.955	37.55/0.967	37.87/0.962	39.04/0.972	**41.08**/**0.986**
	Average	30.88/0.900	33.05/0.935	33.36/0.940	32.44/0.926	33.31/0.945	33.65/0.940	35.75/0.972	**37.51**/**0.983**
30%	1	27.42/0.822	29.37/0.873	29.68/0.883	29.35/0.867	29.69/0.893	29.82/0.881	33.29/0.956	**34.68**/**0.971**
	2	23.91/0.762	25.80/0.916	26.14/0.827	26.46/0.825	26.70/0.842	26.54/0.827	30.59/0.958	**31.94**/**0.974**
	3	28.63/0.845	30.29/0.885	30.68/0.894	30.35/0.891	30.88/0.909	31.02/0.896	33.44/0.959	**35.29**/**0.973**
	4	29.54/0.895	30.72/0.913	31.07/0.919	30.34/0.903	31.31/0.927	31.15/0.920	32.56/0.946	**33.70**/**0.960**
	5	33.32/0.904	34.48/0.931	34.87/0.932	34.68/0.931	35.03/0.944	35.05/0.934	36.47/0.956	**38.32**/**0.976**
	Average	28.56/0.846	30.13/0.904	30.49/0.891	30.24/0.883	30.72/0.903	30.72/0.892	33.27/0.955	**34.78**/**0.971**
40%	1	25.84/0.771	27.13/0.804	27.43/0.818	27.40/0.799	27.70/0.836	27.56/0.812	31.00/0.932	**32.10**/**0.952**
	2	21.04/0.655	23.16/0.718	23.44/0.732	23.93/0.730	23.96/0.740	23.87/0.732	28.68/0.937	**29.17**/**0.953**
	3	26.17/0.777	27.65/0.816	28.07/0.828	28.36/0.839	28.56/0.854	28.38/0.829	31.60/0.939	**32.50**/**0.956**
	4	27.36/0.842	28.69/0.865	29.04/0.874	28.79/0.863	29.42/0.889	29.14/0.874	30.57/0.916	**31.81**/**0.940**
	5	30.72/0.852	32.07/0.889	32.51/0.893	33.02/0.900	32.80/0.911	32.63/0.895	34.23/0.930	**36.12**/**0.962**
	Average	26.23/0.779	27.74/0.818	28.10/0.830	28.30/0.826	28.49/0.846	28.32/0.828	31.21/0.931	**32.34**/**0.953**
50%	1	24.01/0.685	25.07/0.717	25.37/0.731	25.10/0.703	25.54/0.746	25.42/0.722	30.04/0.914	**30.30**/**0.929**
	2	19.65/0.588	20.95/0.626	21.27/0.635	21.75/0.639	21.56/0.634	21.57/0.632	27.23/0.929	**27.74**/**0.939**
	3	24.60/0.713	25.40/0.733	25.96/0.757	26.42/0.772	26.50/0.790	26.12/0.751	30.19/0.921	**31.00**/**0.938**
	4	25.69/0.784	26.82/0.805	27.26/0.819	27.08/0.812	27.63/0.837	27.29/0.816	29.73/0.898	**30.04**/**0.908**
	5	29.26/0.798	29.92/0.839	30.54/0.842	31.19/0.858	31.06/0.867	30.60/0.844	33.54/0.919	**34.39**/**0.947**
	Average	24.64/0.714	25.63/0.744	26.08/0.757	26.31/0.757	26.46/0.775	26.20/0.753	30.14/0.916	**30.69**/**0.932**
60%	1	22.27/0.618	23.21/0.624	23.50/0.642	23.31/0.623	23.53/0.643	23.52/0.631	28.17/0.889	**28.27**/**0.893**
	2	16.75/0.468	18.81/0.518	19.04/0.527	19.36/0.530	18.39/0.481	19.28/0.524	**25.48**/**0.907**	25.34/0.901
	3	21.99/0.607	23.49/0.641	24.11/0.666	24.43/0.684	24.34/0.673	24.22/0.654	28.94/0.904	**29.05**/**0.907**
	4	24.04/0.704	24.94/0.722	25.33/0.739	25.37/0.735	25.55/0.754	25.41/0.736	28.23/0.862	**28.45**/**0.868**
	5	26.44/0.692	28.15/0.770	28.71/0.869	29.54/0.810	29.17/0.796	28.83/0.773	32.08/0.901	**32.88**/**0.924**
	Average	22.30/0.618	23.72/0.655	24.14/0.689	24.40/0.676	24.20/0.669	24.25/0.724	28.58/0.893	**28.80**/**0.900**
70%	1	21.04/0.520	21.41/0.515	21.82/0.532	21.50/0.494	21.17/0.476	21.85/0.518	**26.23**/**0.834**	26.12/0.829
	2	15.50/0.384	16.77/0.404	16.84/0.405	17.04/0.406	15.10/0.312	17.25/0.412	**23.43**/**0.860**	22.29/0.810
	3	20.80/0.520	21.32/0.515	21.99/0.539	22.19/0.549	21.17/0.481	22.09/0.531	26.62/**0.861**	**26.66**/0.853
	4	22.59/0.617	23.16/0.623	23.38/0.636	23.50/0.642	23.12/0.620	23.57/0.634	26.47/0.801	**26.62**/**0.808**
	5	24.86/0.614	25.78/0.683	26.12/0.664	27.06/0.719	26.09/0.663	26.53/0.680	29.82/0.859	**31.47**/**0.884**
	Average	21.00/0.531	21.69/0.548	22.03/0.555	22.26/0.562	21.33/0.510	22.26/0.555	26.51/**0.843**	**26.63**/0.837

**Table 3:** Average running time (in seconds) for eight methods to restore images containing different missing rates and different sizes.

Image Size	Missing Rate	MF	NNM	TNNM	DLMC	NC-MC	LNOP	DMFCNet-1	DMFCNet-2
256 × 256	30%	0.095	4.905	2.974	16.129	5.651	2.384	0.390	0.341
	50%	0.096	4.340	3.455	17.429	3.507	2.165	0.420	0.339
	70%	0.130	3.534	6.816	18.644	2.260	1.649	0.436	0.329
512 × 512	30%	0.288	23.380	12.416	59.581	48.207	9.120	1.763	1.019
	50%	0.199	18.998	17.128	69.528	23.970	8.302	1.661	1.015
	70%	0.116	16.193	18.801	78.087	12.122	7.415	1.769	1.050

**Table 4:** Restoration results of seven methods on five images containing text mask and grid mask.

Mask Type	Images NO.	PSNR/SSIM						
		MF	NNM	TNNM	DLMC	NC-MC	LNOP	DMFCNet-2
Text Mask	1	30.33/0.936	32.37/0.950	32.52/0.952	31.64/0.942	32.42/0.953	32.65/0.953	37.13/0.985
	2	24.80/0.891	28.74/0.926	28.79/0.929	28.47/0.920	29.25/0.932	29.57/0.934	34.63/0.984
	3	30.27/0.930	31.97/0.947	32.49/0.956	32.27/0.948	32.75/0.958	32.57/0.955	35.17/0.985
	4	33.18/0.957	35.16/0.968	35.69/0.971	34.35/0.960	35.54/0.972	35.65/0.971	37.44/0.983
	5	34.30/0.949	37.54/0.975	38.31/0.975	37.96/0.974	38.70/0.979	38.45/0.977	40.82/0.990
	Average	30.58/0.933	33.16/0.953	33.56/0.957	32.94/0.949	33.73/0.959	33.78/0.958	37.04/0.985
Grid Mask	1	29.19/0.899	32.95/0.942	32.96/0.944	33.22/0.928	32.62/0.943	33.20/0.943	37.11/0.983
	2	23.22/0.812	29.30/0.914	29.52/0.918	28.92/0.902	29.75/0.917	30.11/0.919	34.34/0.986
	3	28.41/0.882	33.17/0.946	33.57/0.951	32.44/0.940	33.41/0.954	33.89/0.951	37.67/0.986
	4	31.01/0.933	34.39/0.962	34.59/0.964	33.39/0.952	34.71/0.966	34.72/0.964	36.75/0.980
	5	33.14/0.918	37.45/0.966	37.88/0.967	36.46/0.958	37.78/0.972	38.01/0.968	41.16/0.988
	Average	28.99/0.889	33.45/0.946	33.70/0.949	32.89/0.936	33.65/0.950	33.99/0.949	37.41/0.985

## Data Availability

Not applicable.

## References

[1] Candes E.J., Recht B. (2009). Exact matrix completion via convex optimization. Found. Comput. Math..

[2] Fan J., Chow T.W. (2017). Sparse subspace clustering for data with missing entries and high-rank matrix completion. Neural Netw..

[3] Liu G., Li P. (2016). Low-rank matrix completion in the presence of high coherence. IEEE Trans. Signal Process..

[4] Lu X., Gong T., Yan P., Yuan Y., Li X. (2012). Robust alternative minimization for matrix completion. IEEE Trans. Syst. Man Cybern. Part (Cybern.).

[5] Wang H., Zhao R., Cen Y. (2014). Rank adaptive atomic decomposition for low-rank matrix completion and its application on image recovery. Neurocomputing.

[6] Lara-Cabrera R., González-Prieto A., Ortega F., Bobadilla J. (2020). Evolving matrix-factorization-based collaborative filtering using genetic programming. Appl. Sci..

[7] Zhang D., Liu L., Wei Q., Yang Y., Yang P., Liu Q. (2020). Neighborhood aggregation collaborative filtering based on knowledge graph. Appl. Sci..

[8] Hu Y., Zhang D., Ye J., Li X., He X. (2012). Fast and accurate matrix completion via truncated nuclear norm regularization. IEEE Trans. Pattern Anal. Mach. Intell..

[9] Le Pendu M., Jiang X., Guillemot C. (2018). Light field inpainting propagation via low rank matrix completion. IEEE Trans. Image Process..

[10] Alameda-Pineda X., Ricci E., Yan Y., Sebe N. Recognizing emotions from abstract paintings using non-linear matrix completion. Proceedings of the IEEE Conference on Computer Vision and Pattern Recognition.

[11] Yang Y., Feng Y., Suykens J.A. (2018). Correntropy based matrix completion. Entropy.

[12] Ji H., Liu C., Shen Z., Xu Y. Robust video denoising using low rank matrix completion. Proceedings of the 2010 IEEE Computer Society Conference on Computer Vision and Pattern Recognition.

[13] Cabral R., De la Torre F., Costeira J.P., Bernardino A. (2014). Matrix completion for weakly-supervised multi-label image classification. IEEE Trans. Pattern Anal. Mach. Intell..

[14] Luo Y., Liu T., Tao D., Xu C. (2015). Multiview matrix completion for multilabel image classification. IEEE Trans. Image Process..

[15] Harvey N.J., Karger D.R., Yekhanin S. The complexity of matrix completion. Proceedings of the Seventeenth Annual ACM-SIAM Symposium on Discrete Algorithm.

[16] Lin Z., Chen M., Ma Y. (2010). The augmented lagrange multiplier method for exact recovery of corrupted low-rank matrices. arXiv.

[17] Shen Y., Wen Z., Zhang Y. (2014). Augmented Lagrangian alternating direction method for matrix separation based on low-rank factorization. Optim. Methods Softw..

[18] Toh K.C., Yun S. (2010). An accelerated proximal gradient algorithm for nuclear norm regularized linear least squares problem. Pac. J. Optim..

[19] Cai J.F., Candes E.J., Shen Z. (2010). A singular value thresholding algorithm for matrix completion. SIAM J. Optim..

[20] Chen C., He B., Yuan X. (2012). Matrix completion via an alternating direction method. IMA J. Numer. Anal..

[21] Wen Z., Yin W., Zhang Y. (2012). Solving a low-rank factorization model for matrix completion by a nonlinear successive over-relaxation algorithm. Math. Program. Computn..

[22] Han H., Huang M., Zhang Y., Bhatti U.A. (2018). An extended-tag-induced matrix factorization technique for recommender systems. Information.

[23] Wang C., Liu Q., Wu R., Chen E., Liu C., Huang X., Huang Z. Confidence-aware matrix factorization for recommender systems. Proceedings of the AAAI Conference on Artificial Intelligence.

[24] Luo Z., Zhou M., Li S., Xia Y., You Z., Zhu Q., Leung H. (2015). An efficient second-order approach to factorize sparse matrices in recommender systems. IEEE Trans. Ind. Inform..

[25] Luo Z., Zhou M., Li S., Xia Y., You Z., Zhu Q. (2015). A nonnegative latent factor model for large-scale sparse matrices in recommender systems via alternating direction method. IEEE Trans. Neural Netw. Learn. Syst..

[26] Cao X., Zhao Q., Meng D., Chen Y., Xu Z. (2016). Robust low-rank matrix factorization under general mixture noise distributions. IEEE Trans. Image Process..

[27] Lawrence N., Hyvärinen A. (2005). Probabilistic non-linear principal component analysis with Gaussian process latent variable models. J. Mach. Learn. Res..

[28] Fan J., Chow T. (2017). Deep learning based matrix completion. Neurocomputing.

[29] Fan J., Cheng J. (2018). Matrix completion by deep matrix factorization. Neural Netw..

[30] Nguyen D.M., Tsiligianni E., Calderbank R., Deligiannis N. Regularizing autoencoder-based matrix completion models via manifold learning. Proceedings of the 2018 26th European Signal Processing Conference (EUSIPCO).

[31] Abavisani M., Patel V.M. (2019). Deep sparse representation-based classification. IEEE Signal Process. Lett..

[32] Bobadilla J., Alonso S., Hernando A. (2020). Deep learning architecture for collaborative filtering recommender systems. Appl. Sci..

[33] Zhang S., Yao L., Sun A., Tay Y. (2019). Deep learning based recommender system: A survey and new perspectives. ACM Comput. Surv. (CSUR).

[34] Sedhain S., Menon A.K., Sanner S., Xie L. Autorec: Autoencoders meet collaborative filtering. Proceedings of the 24th International Conference on World Wide Web.

[35] Guo D., Lu H., Qu X. (2017). A fast low rank Hankel matrix factorization reconstruction method for non-uniformly sampled magnetic resonance spectroscopy. IEEE Access.

[36] Huang Y., Zhao J., Wang Z., Guo D., Qu X. (2020). Complex exponential signal recovery with deep hankel matrix factorization. arXiv.

[37] Signoretto M., Cevher V., Suykens J.A. An SVD-free approach to a class of structured low rank matrix optimization problems with application to system identification. Proceedings of the IEEE Conference on Decision and Control (CDC).

[38] Lee D., Jin K.H., Kim E.Y., Park S.H., Ye J.C. (2016). Acceleration of MR parameter mapping using annihilating filter-based low rank hankel matrix (ALOHA). Magn. Reson. Med..

[39] Everingham M., Gool L.V., Williams C.K., Winn J., Zisserman A. (2010). The pascal visual object classes (voc) challenge. Int. J. Comput. Vis..

[40] Everingham M., Eslami S.A., Gool L.V., Williams C.K., Winn J., Zisserman A. (2015). The pascal visual object classes challenge: A retrospective. Int. J. Comput. Vis..

[41] Nie F., Hu Z., Li X. (2019). Matrix completion based on non-convex low-rank approximation. IEEE Trans. Image Process..

[42] Chen L., Jiang X., Liu X., Zhou Z. (2020). Robust Low-Rank Tensor Recovery via Nonconvex Singular Value Minimization. IEEE Trans. Image Process..

[43] Gu K., Zhai G., Yang X., Zhang W. (2014). Using free energy principle for blind image quality assessment. IEEE Trans. Multimed..

[44] Wang Z., Bovik A.C., Sheikh H.R., Simoncelli E.P. (2004). Image quality assessment: From error visibility to structural similarity. IEEE Trans. Image Process..

[45] Liu Z., Luo P., Wang X., Tang X. Deep learning face attributes in the wild. Proceedings of the IEEE International Conference on Computer Vision.

